# Assessing China’s Grain Self-Sufficiency Through National and Provincial Production Dynamics: Implications for Food Security and SDG-2

**DOI:** 10.3390/foods15203759

**Published:** 2026-10-21

**Authors:** Xue Li, Simiao Wu, Jingbing Xu, Xinyang Sun

**Affiliations:** 1 Agricultural Product Processing Institute, Chongqing Academy of Agricultural Science, Chongqing 401329, China; 2 College of Food Science and Engineering, Nanjing University of Finance and Economics/Collaborative Innovation Center for Modern Grain Circulation and Safety/State Key Laboratory Incubation and Development Base for Grain Quality Safety and Nutritional Health, Nanjing 210023, China; 3 Chongqing Institute for Food and Drug Control, Chongqing 401121, China

**Keywords:** China, food self-sufficiency, food security, Sustainable Development Goal (SDG)-2

## Abstract

To better contribute to global progress towards Sustainable Development Goal (SDG)-2, China has been dedicating efforts to developing its capability for ensuring food self-sufficiency and security through a variety of constructive strategies, i.e., legislative cropland protection, sustainable grain production and innovative agricultural technology. As such, the overall objective of this research was to demonstrate China’s situation and performance in developing food self-sufficiency, challenges and opportunities for improving the food security, as well as potential contributions to global progress towards SDG-2. The results demonstrated that maize exhibited the largest sown area and total yield over the past two decades, while rice maintained the highest per unit area yield. Regionally, the Northeast Plain dominated per capita production for maize and soybean (i.e., approximately 220 kg/person for soybean in Heilongjiang), whereas the North China Plain led in wheat production capacities. Despite a stable domestic staple food supply facilitated by robust agricultural policies (i.e., the minimum purchase price policy and high-standard farmland construction), dynamic shifts in dietary patterns and extreme climate events triggered a significant surge in maize and soybean imports between 2019 and 2021. These findings conclude that while China has established a highly resilient foundation for staple grain self-sufficiency, addressing the escalating demand for feed grains through targeted regional optimizations and agricultural innovations remains critical for sustainably fulfilling the targets of global SDG-2.

## 1. Introduction

China, accounting for only 7% of global cropland and freshwater resources, has to provide a sufficient source of food for approximately 18% of the world’s population [1]. To overcome this remarkable challenge, the Chinese government has been working on constructive strategies for the development and improvement of food self-sufficiency across the whole country [1,2,3]. These strategies include a continuous modification of grain policy, i.e., enacting a national law for cropland protection, increasing the sown areas of grains, and ensuring sustainable production of grains, as well as innovative optimization of agricultural technology in terms of grain breeding, cultivation, harvesting and storage [1,2,4,5,6,7,8]. For satisfactory execution of the above strategies, both national and provincial governments in China have been exerting great efforts to strengthen administrative accountability to secure national grain self-sufficiency, contributing to great relief from the pressure on global food security [9].

In addition to the large-sized population, a variety of key factors, i.e., fast urbanization, upgraded dietary patterns, and extreme climate changes, pose outstanding risks to food security in China [8,10,11,12,13,14]. The rapid progress of urbanization over the last two decades has resulted in an incredible shrinkage of cropland size, a remarkable scarcity of freshwater resources, and a great loss of rural labor force [10,12]. Due to the rapid development of urbanization, the dietary structure of Chinese people has been gradually shifting to increased consumption of land-intensive (animal-based) foods, i.e., meat and dairy products [8]. This leads to a significant increase in cropland requirements for foods to the upper limit of domestic cropland resources [8]. Owing to the remarkable influence of extreme climate changes, i.e., long-term heatwaves and multiple typhoon attacks, China has suffered from a severe drought and floods over recent years [14]. This causes a negative impact on China’s food security in terms of grain sowing, growth and yields [15,16]. Particularly, drought-induced damage to the growth of four major crops, i.e., wheat, rice, maize and soybean, was observed over 1.05 million hectares of sown areas in Henan province, accounting for approximately 20% degradation in grain yields across Central China [17].

Although China has been experiencing such a great challenge in ensuring its food security, it is still continuously dedicated to enhancing food production stability and sustainability, as well as improving the food supply and obtaining security, amounting to a considerable contribution to the global pursuit of Sustainable Development Goal 2 (SDG-2), including zero hunger, food sufficiency, nutrition improvement and agricultural sustainability [12,18]. For example, China has been promoting the development of sustainable agriculture through the protection of air, water and land resources, ensuring a long-term and stable increase in the yield of four major crops, i.e., wheat, rice, maize and soybean [11,13,14,19,20].

Numerous studies have evaluated China’s agricultural development, food security policies, and grain production trends. However, most investigations have focused either on national agricultural performance or on specific food security dimensions without integrating long-term production dynamics, regional specialization patterns, and trade dependence within a unified analytical framework. In addition, the implications of increasing feed-grain imports for food self-sufficiency and SDG-2 remain insufficiently explored. Therefore, a comprehensive assessment linking national production trends, provincial heterogeneity, and trade dynamics is needed to better understand China’s contribution to food security and sustainable agricultural development.

Given China’s decent performance in maintaining its food sufficiency and improving its food security, it has good potential to considerably contribute to global progress towards SDG-2. As such, the overall objective of this research was to investigate the feasibility of China’s contribution to the global development of SDG-2 by evaluating the situation and performance of food self-sufficiency development, and also demonstrating the challenges and opportunities for food security improvement. This research has focused on the national and provincial cultivation and production of four major crops, i.e., wheat, rice, maize and soybean, as well as the import and export volume of these above crops. Outcomes from this research will provide abundant information on how China manages sufficient and sustainable production of grains at both the national and provincial levels. This will also benefit us in better understanding China’s food security strategies and its capacity for contributing to the global goal of SDG-2.

## 2. Materials and Methods

This work was conducted to evaluate the capability of China to ensure its food self- sufficiency and security and, in turn, contribute to the global progress towards the Sustainable Development Goal (SDG)-2 [13,18]. Over the twenty years from 2002 to 2021, the sown area, yield, per unit area yield, and import and export volume of four major cereal grains, i.e., wheat, rice, maize and soybean, showed a relatively stable time-dependent evolution of grain production and trade in mainland China, providing solid evidence on China’s good potential to secure a sufficient source of food for its large-size population over a long period of time [4,21].

Over the five years from 2016 to 2021, the average values for sown area, yield, per unit area yield, and per capita grain production of wheat, rice, maize and soybean in thirty-one provinces, i.e., Beijing, Tianjin, Hebei, Shanxi, Inner Mongolia, Liaoning, Jilin, Heilongjiang, Shanghai, Jiangsu, Zhejiang, Anhui, Fujian, Jiangxi, Shandong, Henan, Hubei, Hunan, Guangdong, Guangxi, Hainan, Chongqing, Sichuan, Guizhou, Yunan, Tibet, Shaanxi, Gansu, Qinghai, Ningxia, and Xinjiang, across mainland China, as well as the average production capacity of the four cereal grains, as mentioned above, in seven sowing regions, i.e., North China (Beijing, Tianjin, Hebei, Shanxi and Inner Mongolia provinces), Northeast China (Liaoning, Jilin and Heilongjiang provinces), East China (Shanghai, Jiangsu, Zhejiang, Anhui, Fujian, Jiangxi and Shandong provinces), Central China (Henan, Hubei and Hunan provinces), South China (Guangdong, Guangxi and Hainan provinces), Southwest China (Chongqing, Sichuan, Guizhou, Yunan and Tibet provinces) and Northwest China (Shaanxi, Gansu, Qinghai, Ningxia and Xinjiang provinces), demonstrated regional effects (i.e., climate, environment, technology and policy) on the capacity and per capita level of grain production in China [4,9,14,22].

The data for this work was collected from a variety of national administrations, i.e., National Bureau of Statistics, General Administration of Customs, National Development and Reform Commission and National Food and Strategic Reserves Administration, in China. For example, the National Bureau of Statistics has provided the data source for the sown area, yield, per unit area yield of wheat, rice, maize and soybean across mainland China. According to the data source that is provided by the General Administration of Customs, the National Development and Reform Commission has reported the import and export volume of the four major cereal grains as above. The National Food and Strategic Reserves Administration has provided the data source for investigating the per capita grain production and average production capacity of these four major crops across mainland China.

## 3. Results

### 3.1. Situation and Performance in Developing Food Self-Sufficiency over Decades

**Figure 1:**
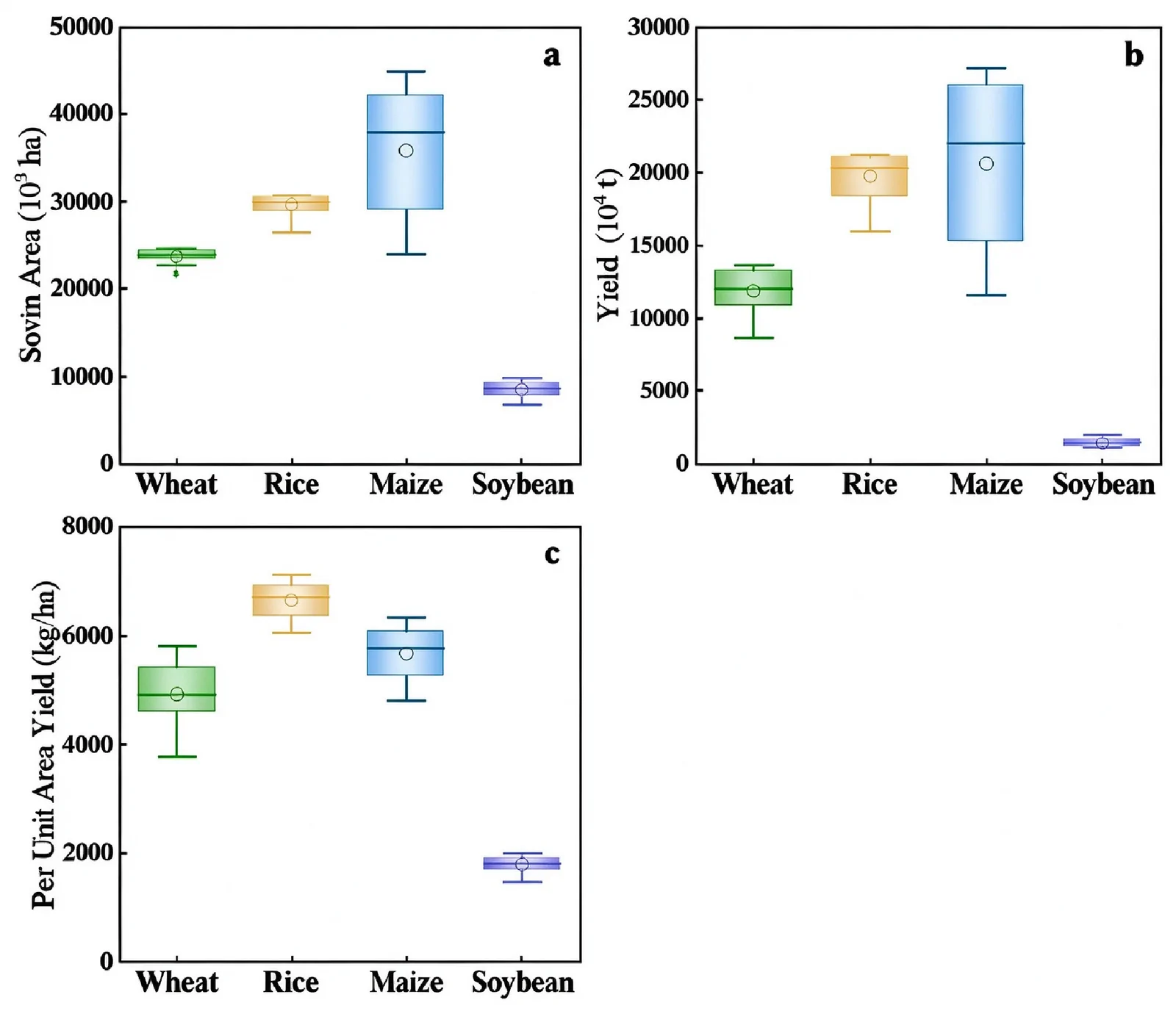
The sown area (**a**), yield (**b**) and per unit area yield (**c**) of wheat, rice, maize and soybean in mainland China from 2002 to 2021.

**Figure 2:**
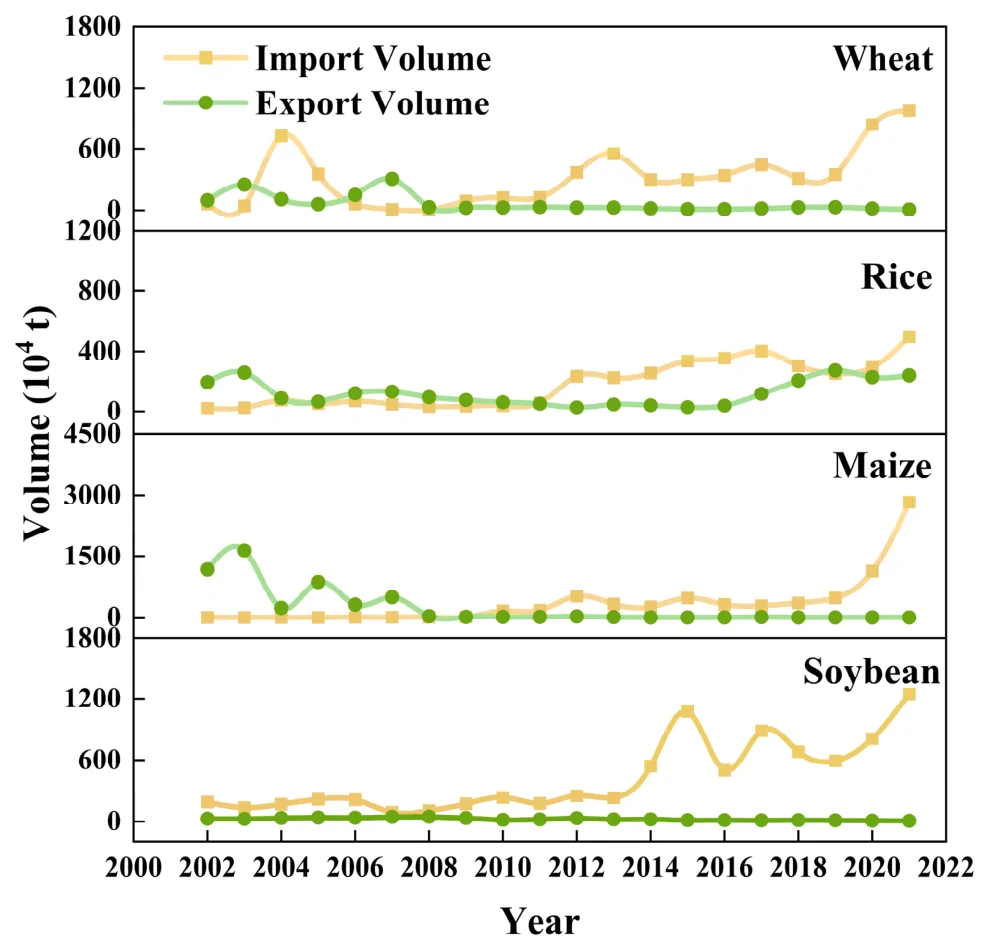
The import and export volumes of wheat, rice, maize and soybean in mainland China from 2002 to 2021.

Over the two decades from 2002 to 2021, China has been working toward a good balance of sowing and yield for four major crops, i.e., wheat, rice, maize and soybean, for better development of its self-sufficiency. The situation and performance in developing China’s food self-sufficiency are illustrated in Figure 1 and Figure 2. To quantitatively assess the interannual variability of crop production from 2002 to 2021, statistical distributions of sown area, total yield, and per unit area yield were analyzed using boxplots. Notably, maize exhibited the highest degree of variability, with its sown area fluctuating dramatically between approximately 24,000 × 10^3^ and 45,000 × 10^3^ ha, and its total yield ranging broadly from roughly 12,000 × 10^4^ to 27,000 × 10^4^ t. This large interquartile range statistically confirms the rapid expansion dynamics of feed grains. In contrast, staple crops such as wheat and rice demonstrated much narrower interquartile ranges, indicating highly stable production scales. Regarding agricultural productivity, rice consistently maintained the highest per unit area yield (averaging approximately 6600 kg/ha with minimal fluctuation), whereas soybean exhibited the lowest productivity, stagnating at roughly 1800 kg/ha across the studied period. According to the average and central values of sown area, yield and per unit area yield for the four crops, the rank of sown area and yield followed the order of maize, rice, wheat and soybean, while the rank of per unit area yield followed the order of rice, maize, wheat and soybean (Figure 1). Compared to the other three crops, maize had the largest sown area and total yield, but its per unit area yield was less than rice’s (Figure 1a,b). Although the sown area and total yield of rice were less than those of maize, it had the largest per unit area yield (Figure 1). The sown area, yield and per unit area yield of wheat were lower than those of rice (Figure 1).

Over the past two decades, a time-dependent variation in the sown area, yield and per unit area yield of four major crops was observed, as shown by the box heights in Figure 1. The sown area and yield of maize had larger variations over years compared to those of other three crops (Figure 1a,b). These fluctuations are not merely random but closely align with the ‘Sickle-shaped region corn structural adjustment’ policy implemented around 2015. This policy aimed to reduce ineffective maize supply in ecologically vulnerable areas, resulting in a temporary but measurable decline in maize sown area between 2016 and 2018, before rebounding due to surging domestic feed demand. Although wheat and rice had a relatively stable sown area over decades, their yield and per unit area yield had obviously time-dependent variations (Figure 1). The temporal trends observed in our updated Figure 1 show a significant and continuous increase in the per unit area yield for rice and wheat from 2004 onwards. This measurable outcome can be directly attributed to major policy milestones, specifically the initiation of the Minimum Grain Purchase Price Policy for rice in 2004 and wheat in 2006, coupled with the complete abolition of the agricultural tax in 2006. These critical policy interventions effectively stimulated farmers’ enthusiasm for grain cultivation, transitioning the yields from a period of stagnation to a statistically significant growth phase. The sown area, yield and per unit area yield of soybean were fixed at a similar level over the time period of twenty years (Figure 1).

Although China has a relatively stable food self-sufficiency, its primary food resources rely on imports. This is demonstrated by comparing the import and export volumes of major cereal grains, i.e., wheat, rice, maize and soybean, from 2002 to 2021 (Figure 2). Over the past two decades, the import volume of these four cereal grains had a larger variation compared to their export volume. From 2002 to 2008, the import and export volumes of wheat had remarkable fluctuations. This fluctuation was also observed for the export of maize. Over the one-decade period from 2011 to 2021, the import volume of soybean experienced an exponential surge, increasing by approximately 700% (representing a Compound Annual Growth Rate of 11.5%). Similarly, a dramatic short-term spike was observed in maize imports, which soared by roughly 480% from 2019 to 2021. These stark quantitative shifts provide robust statistical evidence for China’s growing structural reliance on the global market to meet the escalating domestic demand for feed grains. As demonstrated in Figure 2, China experienced a dramatic exponential surge in soybean and maize imports, particularly after 2014 and peaking between 2019 and 2021. Despite the implementation of domestic support policies such as the Target Price Subsidy Policy for soybeans in 2014 and the Soybean Revitalization Plan in 2019, domestic production remained relatively stagnant. The analytical evidence here reveals a critical gap indicating that domestic policy incentives were insufficient to outpace the massive demand driven by urbanization and dietary shifts towards animal-based proteins, which require massive amounts of soybean and maize for livestock feed. The extreme import spike in 2019–2021 further reflects a combination of post-African Swine Fever herd rebuilding, COVID-19 strategic stockpiling policies, and the Phase One US–China trade agreement.

### 3.2. Challenges and Opportunities for Improving Food Security from Past to Present

**Figure 3:**
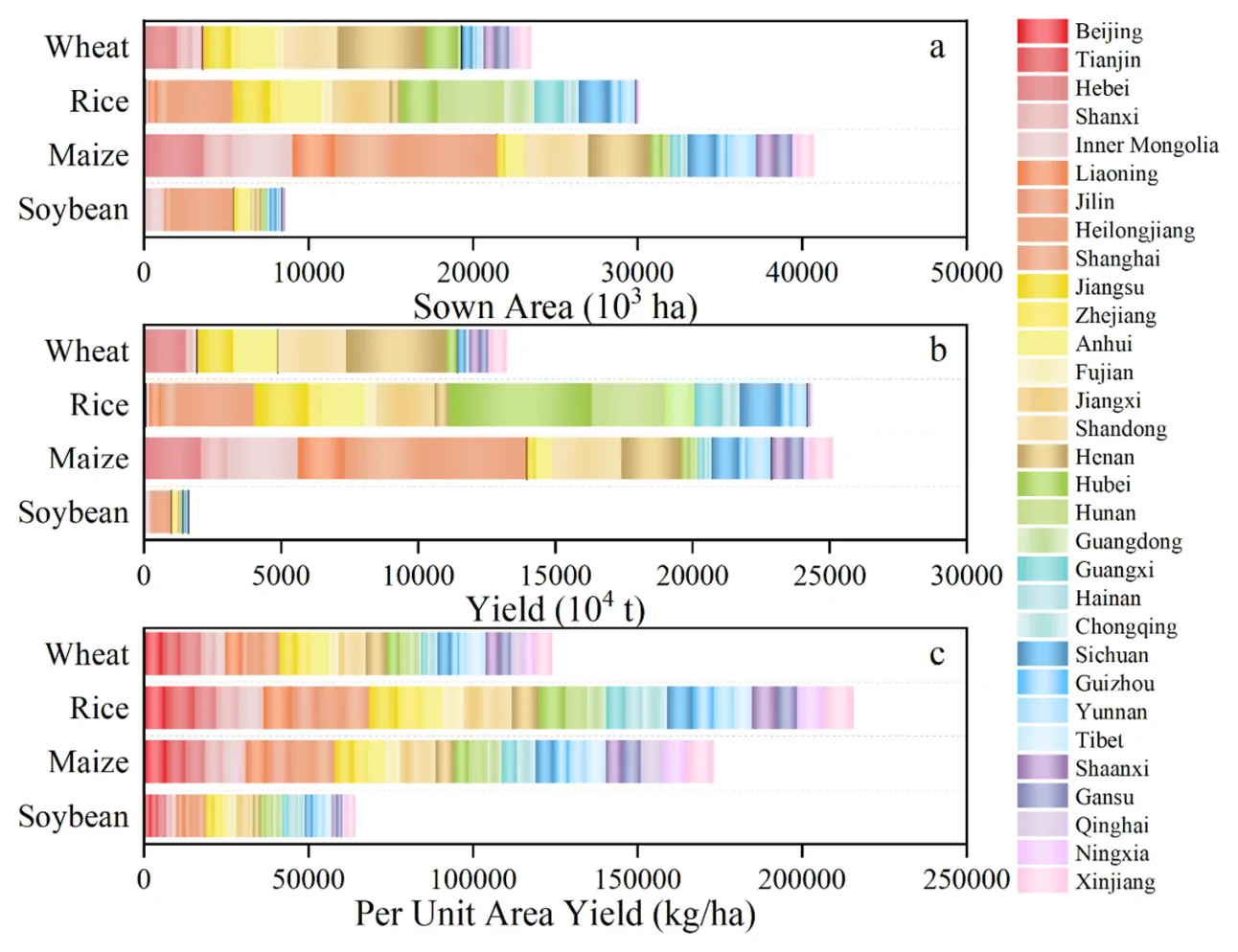
The average values for sown area (**a**), yield (**b**) and per unit area yield (**c**) of wheat, rice, maize and soybean in thirty-one provinces across mainland China from 2016 to 2021.

**Figure 4:**
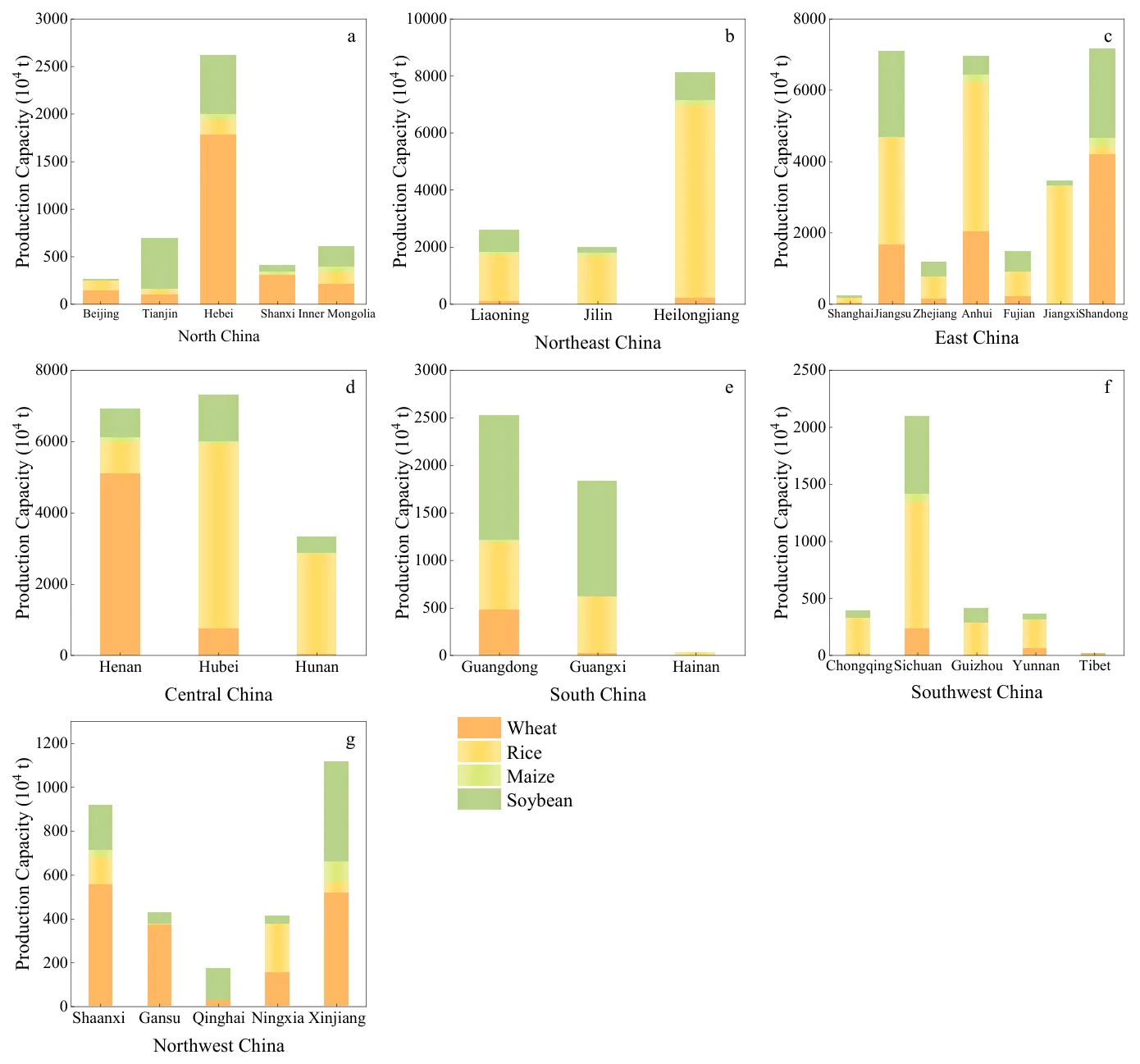
The average production capacity of wheat, rice, maize and soybean in seven sowing regions across mainland China from 2016 to 2021. The sowing region is indicated as (**a**) North China, (**b**) Northeast China, (**c**) East China, (**d**) Central China, (**e**) South China, (**f**) Southwest China and (**g**) Northwest China, respectively.

Over the past five years from 2016 to 2021, the thirty-one provinces across mainland China, also belonging to seven sowing regions, have been dedicating great efforts to sufficient and sustainable development for the sowing and yield of wheat, rice, maize and soybean, to improve China’s food security at a national level. The challenges and opportunities for improving food security in China are illustrated in Figure 3 and Figure 4. Due to differences in geographical and climatical conditions, each province has its suitable sowing crops (Figure 3). Across the thirty-one provinces, Henan, Shandong and Hebei were ranked as first, second and third place, respectively, for having a larger sown area, yield and per unit area yield of wheat crops (Figure 3). However, Sichuan, Guizhou and Yunnan had a smaller sown area, yield and per unit area yield of wheat crops (Figure 3).

Over a wide range of provincial locations, Hubei, Hunan, Jiangsu, Zhejiang and Anhui were seen to have a relatively larger sown area and yield of rice crops (Figure 3), while Jiangsu, Zhejiang and Anhui tended to have a higher per unit area yield of rice crops compared to Hubei and Hunan (Figure 3). Shaanxi, Gansu, Qinghai and Ningxia in Northwest China had much smaller sown area, yield and per unit area yield of rice crops compared to other provinces (Figure 3). Both maize and soybean had larger sown areas, yields and per unit area yields in the provinces of Liaoning, Jilin and Heilongjiang (Figure 3). In conclusion, China’s grain production exhibits a high degree of spatial agglomeration. Specifically, staple crop cultivation is heavily concentrated in a few core geographic hubs: wheat production is strictly dominated by the North China Plain (i.e., Henan and Shandong), whereas maize and soybean are highly dependent on the Northeast region (notably Heilongjiang, which accounts for the vast majority of soybean yield). Meanwhile, rice production is jointly sustained by the Middle–Lower Yangtze Plain and the Northeast. This distinct spatial heterogeneity emphasizes that maintaining the agricultural resilience of these few core provinces is critical to the overall stability of national food security.

The thirty-one provinces across mainland China have been classified into seven sowing regions according to their geographical location. Over the five-year period of 2016–2021, the average production capacity of wheat, rice, maize and soybean varied due to their sowing regions. This is illustrated in Figure 4. Across the seven sowing regions, Northeast, East and Central China had larger production capacities of these four cereal grains (Figure 4b–d). However, a lower production capacity of these cereal grains was observed for Southwest and Northwest China compared to the other sowing regions (Figure 4f,g). The comparative analysis reveals a spatial polarization. The core agricultural hubs, specifically Central China (i.e., Henan, Hubei) and Northeast China (i.e., Heilongjiang), exhibited overwhelmingly superior production capacities, with peak regional outputs reaching approximately 7000 × 10^4^ to 8000 × 10^4^ t. In stark statistical contrast, the production capacities in Northwest and Southwest China were significantly lower, generally constrained below 1200 × 10^4^ t and 2500 × 10^4^ t, respectively. Furthermore, structural comparisons indicate clear regional specialization. Central and East China demonstrate a statistically balanced co-dominance of wheat and maize (i.e., Henan and Shandong), whereas the Northeast is structurally dominated by a combination of rice and maize. This quantitative and structural comparison firmly corroborates the extreme spatial imbalance and functional specialization of China’s food production system.

### 3.3. Potential Contribution to Global Progress Towards SDG-2

**Figure 5:**
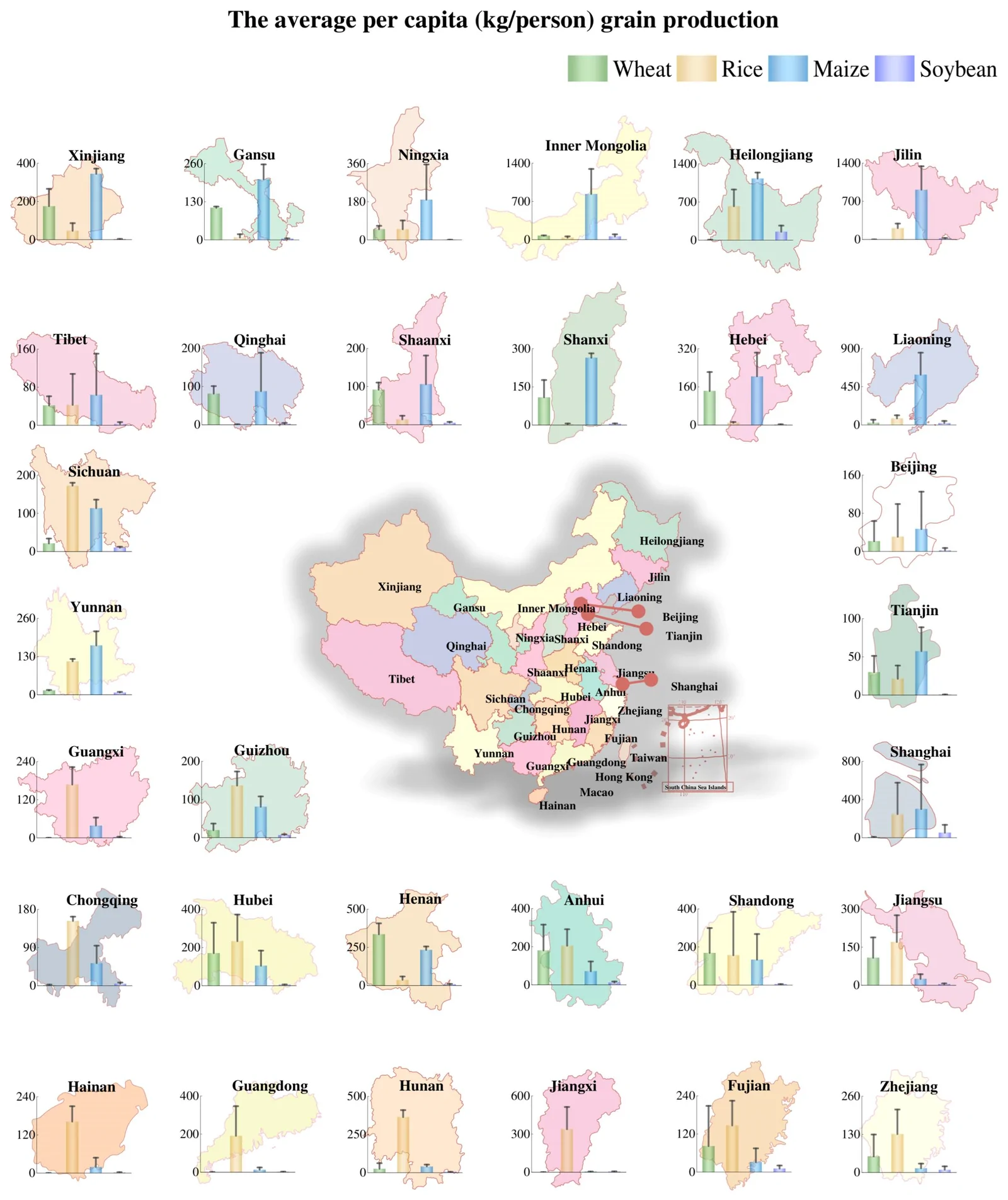
The average per capita grain production in thirty-one provinces across mainland China from 2016 to 2021. Grains include wheat, rice, maize and soybean.

While the fundamental production and trade statistics demonstrate China’s capacity for food self-sufficiency, these quantitative metrics must be explicitly evaluated against the multidimensional targets of SDG-2. Specifically, the substantial per capita grain production observed in the Northeast and North China Plains provides a resilient caloric foundation that directly aligns with the primary goal of ending hunger. Concurrently, the continuous increase in the per unit area yield of staple crops, particularly rice and wheat, serves as empirical evidence for improving agricultural productivity, achieved through advanced cultivation techniques rather than sheer land expansion. Furthermore, the exponential growth in soybean and maize imports, although posing a challenge to domestic self-sufficiency, intrinsically corresponds to the broader target of reducing malnutrition. This structural trade shift accommodates a nationwide dietary transition toward higher animal-based protein consumption, thereby enhancing overall nutritional diversity and dietary quality beyond basic caloric survival. In addition to the remarkable outcomes from the development of China’s food self-sufficiency and security, China also has strong production capacity of four major cereal grains, i.e., wheat, rice, maize and soybean, further revealing its potential contribution to global progress towards SDG-2. This is illustrated in Figure 5. Since the production capacities of wheat, rice, maize and soybean and the population size are differentiated between provinces in China, the average per capita production for these major grains varied according to its sowing location (Figure 5). Over the past five years from 2016 to 2021, four provinces in the Northeast Plain, i.e., Inner Mongolia, Heilongjiang, Jilin and Liaoning, had larger average per capita maize production, with a range from 600 to 1200 kg per person. The largest average per capita maize production was observed in Heilongjiang compared to other provinces, while Hebei, Henan and Shandong in the North China Plain had larger average per capita wheat production, i.e., 150 to 350 kg per person. Particularly, Henan had the largest average per capita wheat production across mainland China. Compared to the other provinces, Hubei, Hunan and Jiangsu in the Middle–Lower Yangtze Plain had a larger average per capita rice production, with a range of 250–380 kg per person. Unlike the other thirty provinces, where the average per capita soybean production was less than 100 kg per person, Heilongjiang had the largest average per capita soybean production, i.e., approximately 220 kg per person.

## 4. Discussion

By comparing the sown area, total yield and per unit area yield of four major cereal crops, maize is considered the primary crop sown over the last two decades from 2002 to 2021. This is due to the remarkable role of maize, which acts as a good source of raw material for the production of foods, feedstuffs and energy [13]. Rice shows the largest per unit area yield among these cereal crops due to the continuous development of sowing techniques and the positive influence of governmental policies [16,23]. This has also been interpreted as being due to the significant popularity of rice, which serves as the essential staple food in the daily diet of the majority of Chinese people [21,24].

A smaller sown area, yield and per unit area yield of wheat compared to those of rice have been interpreted as being due to the lower popularity of wheat, which mainly serves as a staple food for people from northern China [25]. Although the domestic yield of wheat is capable of meeting the basic needs of wheat products for Chinese people, it is still challenging to get access to strong wheat cultivars with good quality due to technical limitations in wheat breeding [25,26]. To obtain well-known wheat cultivars with stronger gluten proteins that are suitable for bakery manufacturing, China has to rely on international imports of these wheat cultivars, and also develop domestic wheat cultivars that have a higher content of stronger gluten proteins with a balanced ratio of glutenins to gliadins [27]. The lowest sown area, yield and per unit area yield of soybean are interpreted as being due to its limited popularity as a source of edible oils that are consumed by people from northeast China [4].

A larger variation in the sown area and yield of maize has been observed, indicating a significant development of maize sowing from the past to present that is affected by the development of governmental policies and sowing techniques [7,16,21,23]. A significant variation in the yield and per unit area yield of wheat and rice over the last two decades has been seen due to the impacts of technique development and climate change [25,26]. A relatively fixed sown area, yield and per unit area yield of soybean have been observed due to the stable supply of soybeans from international import, which limits the development of local soybean sowing [28].

A larger variation has been seen in the import volumes of four major cereal grains compared to their export volumes over the past two decades, indicating that the government has relatively stable control over the export of cereal grains [5,7,18]. A significant fluctuation has been observed in the import and export volumes of wheat, as well as the export of maize, over the six-year period of 2002 to 2008. This phenomenon is due to China becoming a member of the World Trade Organization (WTO) in 2001, leading to significant changes in its global trade policy for cereal grains after this year [5,28,29]. The policy evolution has been observed to significantly affect the total factor productivity (TFP) of three major grains, i.e., wheat, rice and maize, which is positively correlated with grain production in China [7]. A remarkable fluctuation has also been observed in the import volumes of wheat and soybean over the one-decade period of 2011–2021. This has been interpreted as being due to the lower sown area and yield of these cereal grains domestically, which causes a higher dependence of their supply on global import [30]. Over a three-year period from 2019 to 2021, an obvious increase in the import volume of four major cereal grains, i.e., wheat, rice, maize and soybean, has been observed. This is probably due to the unexpected influence of the global pandemic on food security, which led the Chinese government to implement positive policies for securing domestic supply of staple foods [29,31].

To better contextualize China’s food security status, it is essential to compare its agricultural endowment with those of other major agricultural nations. Unlike the United States or Brazil, which possess abundant arable land and act as major net exporters, China must support approximately 18% of the global population with only 7% of the world’s arable land and freshwater resources [32,33]. Despite achieving basic self-sufficiency in staple grains (wheat and rice), China remains in a position where it must heavily import feed grains (soybeans and maize). This high reliance on imports is not due to a decline in domestic production capacity, but rather a structural deficit driven by rapid urbanization and an upgraded dietary pattern. As the population consumes more land-intensive, animal-based foods (meat and dairy), the demand for livestock feed has surged beyond the upper limits of domestic ecological carrying capacity, necessitating large-scale imports to fill the gap [34].

To mitigate this structural deficit and ensure long-term self-sufficiency, several actionable strategies and international good practices should be implemented. First, from a technological perspective, accelerating the innovation of seed breeding (i.e., drought-resistant and high-yield traits) is crucial to maximizing the output of limited arable land. Second, adopting alternative feed strategies, such as promoting low-protein diets for livestock and utilizing microbial proteins or insect-based proteins, can significantly reduce the structural dependency on imported soybeans [34]. Finally, learning from international best practices in demand-side management, developing a coherent policy framework, implementing national campaigns to reduce food waste and guiding the public toward more sustainable, plant-forward dietary structures will effectively alleviate the pressure on domestic grain production and enhance overall food security [35,36].

Over the five-year period of 2016–2021, a larger sown area, yield and per unit area yield of wheat crops have been observed for Henan, Shandong and Hebei compared to the other provinces across mainland China. The three provinces above are located in the North China Plain, which has a larger number of flat areas, lower level of precipitation, and longer duration of sunshine, which favor the growth of wheat crops [37,38,39], while a smaller sown area, yield and per unit area yield of wheat crops have been seen for Sichuan, Guizhou and Yunnan over the past five years from 2016 to 2021. This is due to their location in Southwest China, which has a larger number of hilly areas, higher level of precipitation, and longer duration of sunshine, which delays or even inhibits wheat growth [26,38,39].

A relatively larger sown area and yield of rice crops have been observed for Hubei, Hunan, Jiangsu, Zhejiang and Anhui over the five-year period from 2016 to 2021. This has been interpreted as due to the locations of these five provinces in the Middle–Lower Yangtze Plain, which has larger-size flat areas with a sufficient source of freshwater, contributing to good growth of rice crops [39,40]. As such, the sown areas for cereal grains have to be guaranteed in the Middle–Lower Yangtze Plain by considering its good availability of land–water resources [37]. Jiangsu, Zhejiang and Anhui have been seen to have a higher level of per unit area yield compared to Hubei and Hunan. This indicates higher-efficiency and more advanced sowing techniques for rice crops that are conducted by Jiangsu, Zhejiang and Anhui [23,29]. A significantly smaller sown area, yield and per unit area yield of rice crops have been observed for Shaanxi, Gansu, Qinghai and Ningxia due to a shortage of freshwater areas and low level of annual precipitation in these above provinces in Northwest China [37,38]. Over the past five years from 2016 to 2021, Liaoning, Jilin and Heilongjiang have been seen to have a larger sown area, yield and per unit area yield of maize and soybean compared to the other provinces across mainland China. These three provinces are located in the Northeast Plain, which is well known for a vast area of fertile dark soil, favoring the growth of rice and soybean with a desirable overall quality [31,38].

Over the five-year period of 2016 to 2021, stronger production capacities of four major cereal grains have been observed for Northeast, East and Central China across seven main sowing regions. This is due to the locations of the above sowing regions in the Northeast Plain, Middle–Lower Yangtze Plain and North China Plain, respectively, with a variety of favorable conditions, i.e., large-size flat area, fertile soil, suitable precipitation, and sufficient distribution of freshwater, for good growth and yield of the above cereal crops [31,38]. The pattern of grain production in China has been observed to shift towards the sown areas in northern China, i.e., the Northeast Plain and North China Plain, due to a significant increase in the precipitation over the 400 mm annual precipitation line for these sown areas [40]. Southwest and Northwest China have been seen to have a weaker production capacity of these cereal grains compared to other sowing regions. This has been interpreted as being due to these two regions having a series of complex geographical and climatical conditions, i.e., a large number of mountainous and hilly areas, unpredictable precipitation, and even a lack of freshwater in Northwest China, which cause great challenges in securing the growth and yield of cereal crops to a decent extent [31,38,39].

According to Chinese history from 1959 to 1961, 23 to 30 million people were suffering from famine due to insufficient yields of cereal grains, which were incapable of feeding the total population [24]. To solve this issue and, in turn, pursue the Sustainable Development Goal (SDG)-2 of zero hunger, the government, scientists and industry have been collaborating to modify policies, processing techniques and measures to achieve a good enhancement in the yield and production capacity of cereal grains [5,18,21,30].

Over the past five years from 2016 to 2021, the provinces in the Northeast Plain had the highest level of average per capita maize production due to its favorable sowing conditions (i.e., larger-size flat areas, fertile soil and suitable precipitation), large-scale advanced sowing techniques and smaller-sized population [23,38]. In particular, Heilongjiang had the largest average per capita production for both maize and soybean. This was also due to governmental policies that encourage the development of cereal crop sowing in this province, with good consideration of its unique advantages [29].

The per capita wheat production in China has been considered to primarily depend on climate change, which may cause an increase in the total wheat production [25]. A larger average per capita wheat production has been observed for the provinces in North China Plain, which has suitable climate and environmental conditions, i.e., a larger number of flat areas, lower-level annual precipitation, and a longer duration of sunshine, for better growth of wheat crops [25,38,39]. Since a series of continuous collaborations have been conducted between government, research associations and industries, Henan not only has the largest average per capita wheat production, but it has also been developing wheat cultivars with a higher gluten strength and better processing performance [25,41]. Several provinces in the Middle–Lower Yangtze Plain have demonstrated a larger average per capita rice production due to their great natural conditions, i.e., a larger number of flat areas and sufficient sources of freshwater, which is considered the optimal sowing location for rice crops [37,38].

Beyond productivity and nutritional improvements, China’s agricultural evolution critically addresses the SDG-2 mandates of ensuring sustainable food production systems and increasing resilience to climate-related risks [42]. The strict implementation of national land-protection policies, coupled with extensive high-standard farmland construction across the Middle–Lower Yangtze Plain and North China Plain, represents a systemic transition toward sustainable land management that aims to decouple yield growth from ecological degradation [4]. Moreover, as extreme weather events such as prolonged heatwaves and severe flooding increasingly threaten crop stability in traditionally fertile regions, strategic regional optimizations serve as vital adaptive mechanisms. By gradually consolidating core production capacities in regions with optimized water–land matching and resilient agricultural infrastructure, China not only mitigates immediate agronomic vulnerabilities but also embeds long-term climate resilience into its national food security framework [14,22,43]. This multifaceted approach underscores a comprehensive and structural contribution to the global SDG-2 agenda.

**Figure 6:**
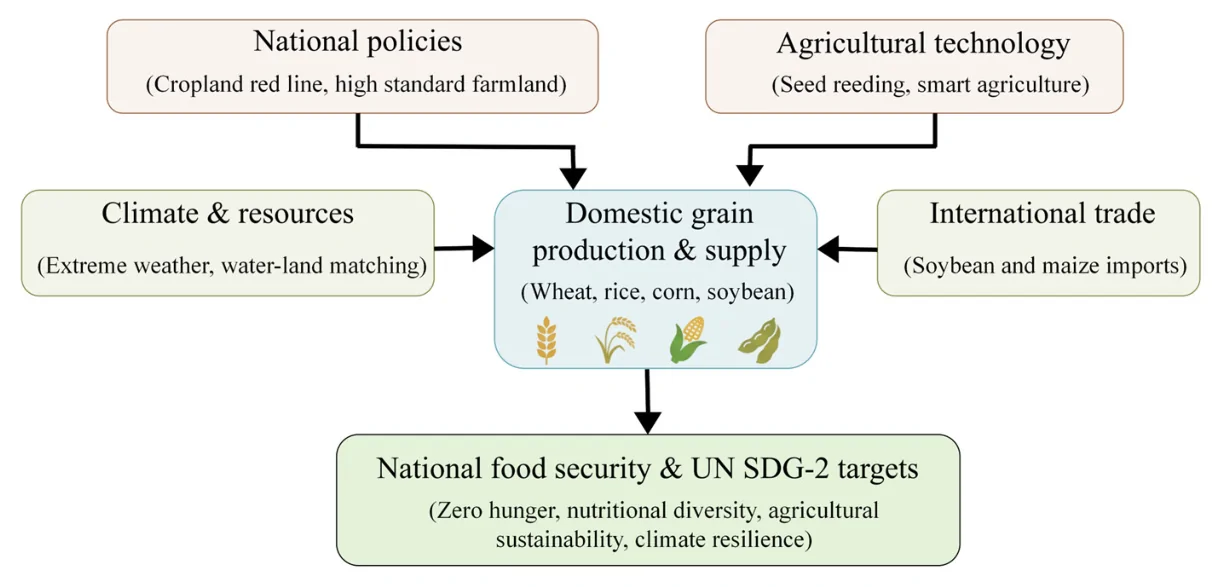
Conceptual map of the multidimensional factors and conditions influencing China’s grain production, food self-sufficiency, and progress toward UN SDG-2.

Viewed through a strategic SWOT (Strengths, Weaknesses, Opportunities and Threats) framework, the current state of China’s food security presents clear multidimensional characteristics. The primary strength lies in the sustained high self-sufficiency and robust production capacity of staple crops (wheat and rice). However, a critical weakness is the structural vulnerability associated with the massive import reliance on feed grains (soybean and maize). Moving forward, strong policy incentives and technological advancements in agricultural resilience offer significant opportunities to mitigate these issues. Conversely, the extreme spatial concentration of production and the northward shift of the agricultural center pose ongoing threats related to regional ecological pressure and logistical vulnerabilities. To visually synthesize these multidimensional interactions, a comprehensive conceptual map illustrating the driving factors and systemic pathways toward China’s food security and SDG-2 is presented in Figure 6.

## 5. Conclusions

This study evaluated long-term changes in grain production, productivity, and trade in China between 2002 and 2021. The results indicated that China maintained strong self-sufficiency in staple grains through sustained productivity growth and regional production specialization. Nevertheless, increasing dependence on imported soybean and maize suggests an emerging structural challenge associated with feed-grain demand. While China’s agricultural system demonstrates considerable resilience, future progress toward SDG-2 will depend on strengthening feed-grain production, enhancing climate resilience, reducing regional vulnerabilities, and maintaining sustainable agricultural productivity.

## Data Availability

The original contributions presented in this study are included in the article. Further inquiries can be directed to the corresponding author.
